# Understanding students’ motivations for participating in a mindfulness course: a qualitative analysis of medical students’ views

**DOI:** 10.1186/s12906-023-03949-2

**Published:** 2023-05-20

**Authors:** Cloé Brami, Serge Sultan, Léonore Robieux, Marie-Aude Piot, Honorine Gartili, Franck Zenasni

**Affiliations:** 1 LaPEA - Université Paris Cité and Univ Gustave Eiffel, LaPEA, Boulogne- Billancourt, F-92100 France; 2 GIS Théorie et Pratiques du Care, Université Paul Valéry, Montpellier, France; 3 Centre Hospitalier Universitaire Sainte-Justine, Québec, Montréal Canada; 4 Department of Psychology, Université de Montréal, Montreal, QC Canada; 5 Laboratory of Psychopathology and processes of change, Université Paris 8, LPPC, Saint Denis, F- 93000 France; 6 Necker Enfants malades hospital, Child and adolescent psychiatry unit, Université de Paris-Cité, AP-HP, CESP, USQV Paris-Saclay, Paris, Inserm 1018 France

**Keywords:** Soft skills, Mindfulness, Medical students, Mental health, Education, Integrative medicine

## Abstract

**Objectives:**

Improving medical students’ wellbeing and empowerment through curricular activities is a topic of interest worldwide. Mindfulness-based interventions (MBIs) are increasingly implemented in medical education often as part of elective courses. To better understand training outcomes and adjust curriculum to students’ needs, we will explore why will medical students participate in meditation-based education?

**Methods:**

We analyzed 29 transcripts from the first session of an 8-week MBSR program offered to medical students in French. Transcripts were coded and analyzed using a qualitative content thematic analysis and the constant comparison method.

**Results:**

Analyses resulted in three themes describing students’ motivation: (1) Medical education and the physician’s role, i.e. improving interpersonal skills, acquiring skills oriented toward a more integrative medicine, being more productive in a highly competitive context. (2) Caring for my health i.e. aiming at stress reduction, emotion regulation, and improving self-compassion. (3) A quest for meaning, i.e. optimizing meaning of care, and meaning of life.

**Conclusion:**

The results highlight the congruence between the perceived motivations and the evidence on the effect of mindfulness on self-care, the development of humanistic medical skills, and the meaning of care. Some findings raise the issue of the limits of using mindfulness to enhance one’s productivity. Notably, participants articulated the need for self-care as in mindfulness training, with the ability to care for others.

**Supplementary Information:**

The online version contains supplementary material available at 10.1186/s12906-023-03949-2.

## Introduction

Medical students are particularly at risk for mental health problems and academic burnout worldwide [1–3], including in France [4–6]. Over the decades, research has been focused on studying the efficacy of mindfulness-based interventions such as the mindfulness-based stress reduction program (MBSR) [7,8]. In fact, in the medical education context, improving medical student well-being through educational activities is a topic of interest to promote emotional awareness, self-care and provide resources to deal with distress [9–12]. Mindfulness-based interventions (MBI) are increasingly being implemented in medical education as part of courses focused on student well-being [12–15]. So far mindfulness was found to favor mental well-being in medical students with positive outcomes including stress reduction, resilience, quality of life, anxiety, and depression [9,13,14,16,17].

Although much work has been done on how and when these electives courses should be offered in medical school, very little is known on the view of users. Moreover, since the impact of course, including the transformative aspect of mindfulness [18–20], depends on expectations and motivation, it is necessary to collect this data. Kabat-Zinn, for instance, asserted that *one’s motivation sets the conditions for personal growth and the possibility of change* [21]. According to Shapiro et al., qualitative study, on the motivation of meditation practitioners, the motivation to practice *shifts along a continuum from self-regulation to self-exploration, and finally to self-liberation* as the meditators continue to practice [22]. As in many non-pharmacological interventions, this study found a fit between the training outcome (e.g. relaxation) and one’s motivation to participate (e.g. to reduce stress).

From a psycho-educational perspective, ‘motivation to learn’ has been described as a student’s ‘energy and drive to learn, work effectively and achieve to their potential’, in addition to the behaviors associated with this energy and drive [23]. Hence, understanding students’ motivation to train in a mindfulness course would allow us to (1) better understand the findings on the impact of mindfulness and (2) help medical universities tailor the curriculum to students’ needs. Importantly, such data is systematically collected (but never analysed) within the 8-week MBSR program. This happens during the first session designed to explore the motivations of participants for being there [24,25].

Our study will use this clinical data from MBSR training to investigate the following question: why will medical students participate in meditation-based education? In this study, we wished to uncover their motivations as these could be key to explain future adherence and effects of MBI.

## Methods

In order to meet these objectives, the present study will analyze the transcript dialogue collected during the first session of an MBSR training proposed to medical students. Within the first session of the program, the dialogue period is framed to be a collective semi-directive interview focused on motivations to participate.

### Inclusion criteria and student recruitment

The MBSR program was offered between November 2019 to January 2020 to medical students as an elective course. The courses took place in a research center affiliated to the Université Paris Cité. Potential participants were informed about this class through social media. They were then included after participating in an orientation session regarding the modalities of the program and the research. Students were informed that their participation would not affect their academic and clinical training. The class was offered as a pilot project to any level of medical training from the beginning (years 2) to the end (residency). Inclusion criteria were: (1) attendance to the orientation session, (2) studying in a medical school in Paris (year 2 to residency), (3) studying in Paris, (4) minimal age of 18, (5) volunteers, (6) never participated in an MBSR program before, (7) no mental health disorders as screened by a medical doctor during orientation session (psychotic symptoms, active substance dependance, suicidal ideations). The study was approved by an ethical committee (N°IRB : 00012019–34). The course was free of charge and participants did not receive financial compensation for their participation.

### MBSR program

Students signed up to attend the standard MBSR program, including 8-week group sessions of 2.5 h and a full day silent retreat [26]. Each session included meditation practices, group discussions, and teaching. Group discussions consisted of a dialogue period between teacher and student. The training was conducted in French by experienced mindfulness trainers (SM, CB) who all met the requirements of the good practice certified by Brown University. To ensure the integrity of the program, one of the instructors (SM) had more than 8 years of experience. As the study is qualitative, we wish to recognize the importance of our personal backgrounds and how they may play a role in the results (criterion of transparency, Appendix 1).

### Data collection

Datas was collected during the first session of the MBSR program. This session was designed to explore, in depth, participants’ motivation to participate [27]. At the beginning of the class, after a short meditation practice, participants were all asked to answer an open-ended question “Why are you here?“. Teachers ask students to spontaneously observe any responses that emerge with a “non-judgmental” attitude described in the practice [21]. Then, each participant was invited to share their answers. Importantly, sharing one’s motivation was not mandatory and students could opt out from this. All participants responded in turn using short sentences for a period of 2–10 min. Teachers were allowed to rephrase the answer at times to ensure correct understanding. The recording of the dialogue was then fully transcribed and anonymized before analysis.

### Data analysis

Thematic analysis is the most common form of analysis in qualitative research [28]. Thematic analysis consists of *“systematically identifying, grouping and, subsidiarily, examining the discourse of the themes addressed in a corpus”* [29]. Thematic analysis is performed through a process of coding to create established, meaningful patterns. This method can be applied to any type of signifying material [30]. It pinpoints, examines, and records patterns (or “themes”) within data and the themes become the categories for analysis. To perform the data analysis in a structured method, we used the six steps proposed by Braun et al. [28] At first, the researchers independently (CB and HG) read (*step 1: familiarizing with data*) and coded the transcribed text of each interview (*step 2: generating initial codes*), and stayed semantically close to the participants’ wording. In a second step, we started the process of grouping codes into categories or sub-themes after coding the first transcript (*step 3: searching for themes*). During this inductive coding process, the researchers discussed the findings until mutual agreement was achieved (*step 4: reviewing themes*). Then, connections between categories were then examined to form overarching themes (*step 5: Defining and naming themes*). The process of coding and categorizing were discussed at a research meeting with all authors. The constant iteration of these steps led to a deep understanding of the student’s motivation to learn mindfulness. The Consolidated criteria for Reporting Qualitative research (COREQ) [31] were applied in reporting the results (*step 6: Producing the report*).

### Ethical considerations

Participants received complete written information about the scope of the research, the identity and affiliation of the researchers, the possibility of withdrawing from the study at any point, and confidentiality. All participants provided written informed consent and the study was approved by the ethics committee (IRB: 2019-92).

## Results

Twenty-nine students voluntarily joined the MBSR class and attended the first session. 62% were undergraduate (beginning, n = 4(15%); Clerkship, n = 12(41%)), 45%(n = 13) were residents (n = 13), 65% (n = 20) were women. Mean age was 25.9 years old. 3(10%) students have already meditated. Participant characteristics are shown in Table 1.

**Table 1:** Characteristics of 29 participants to the MBSR program

Gender n (%)	
Men	9 (35%)
Women	20 (65%)
Age, mean (SD)	25,9
University level, n (%)	
Beginning (2 to 3 years)	4 (15%)
Clerkship (4 to 6 years)	12 (41%)
End – residency (7 to 9 years)	13 (45%)
Medical school	
Paris 5 - Descartes	11 (42.3%)
Paris 6 - Sorbonne Université	7 (26.9%)
Paris 7 - Diderot	7 (26.9%)
Other	1 (3.8%)
Have been meditated already with phone app.	3 (10%)

**Fig. 1:**
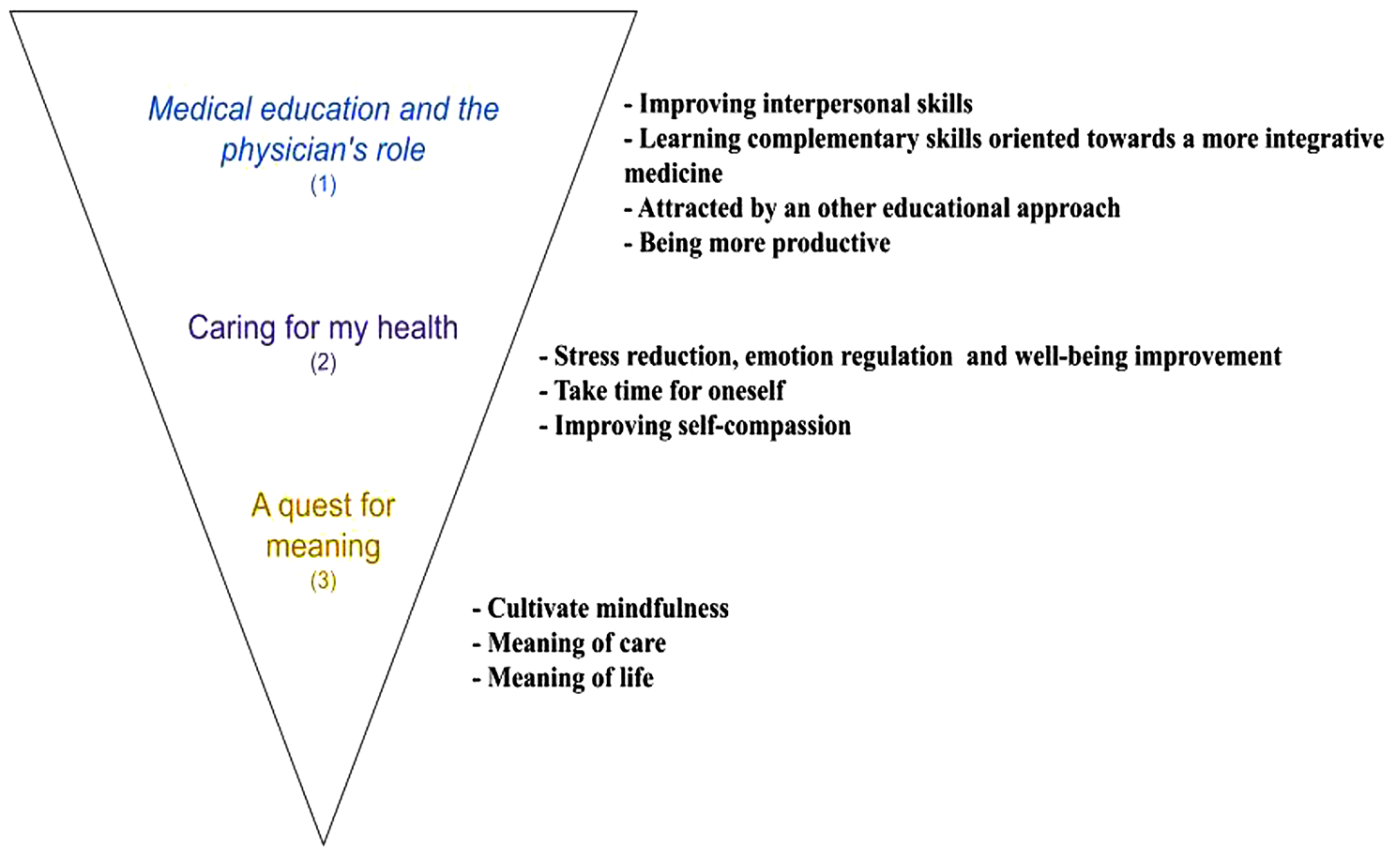
Themes and subthemes of the analyses of medical students’ motivation to participate in a MBSR class

We identified three overarching themes based on views shared by students on their motivations to participate in the mindfulness-based class. These themes varied on their nature of politically correctness, as well as their personal nature. The first theme gathers motivations relevant to medical training and the professional role of the physician. The second theme gathers codes pertaining to the need to take care of one’s own health. The third theme gathers reports on searching meaning in medical practice and life in general. Themes and subthemes are summarized in Fig. 1. A detailed account of codes is available in Appendix 2.

### Medical education and physician’s role

First, participants reported motivations related to medical education in line with their commitment to their studies. Participants wished to improve their skills. Within this theme, we were able to identify four subthemes.

#### Improving interpersonal skills

Participants expressed a need to develop interpersonal skills allowing them to develop more meaningful relationships with their patients. They mentioned the lack of resources related to the communication skills they had access previously, before engaging in the MBSR program.

*“I thought I liked being in a relationship, a real relationship with the patients and the people we were talking to(E7)”.*

*“I hope to be able to find the right words a little more and to have a sympathetic ear(E24)”.*

They wanted to learn how to develop a relationship being more centered on the patient’s needs. At the same time, they wished to acquire skills allowing them to step back from the overwhelming emotions they reported in the healthcare relationship. Some shared experiences of conflict, unpleasantness, or emotional contagion during care. The need to better control one’s own emotion, such as aggressiveness or emotional variability, was also underlined.

*“Why I’m really really here, I thought it might teach me to understand my emotions(E4)”.*

#### Learning complementary skills oriented towards a more integrative medicine

Some participants would like to learn more holistic dimensions of care. They wished to participate in the MBSR program to learn about meditation as an element of a more integrative medicine that takes into account the body-mind connection. They also emphasized the desire to integrate meditation into their care activities, and therefore the need for training.

*“I like to test everything I offer my patients so I find it interesting to know what we’re talking about, so that was the primary reason(E21)”.*

#### Attracted by an other educational approach

Some participants reported the too theoretical, perhaps too serious learning methods they experienced in their medical studies. Attending such a program seemed also a way to enjoy a new experiential learning experience. They wished to experience joy, desire, and enthusiasm and discover a new activity.

*“So there you go, as I was saying to M…, I was excited to do this (laughs)(E1)”.*

They also reported the desire to be in a group and learn with and from others. Indeed, they looked for group training, insisting on fostering social bonds.

*“I wanted to __ not just to learn something but that __ or to learn things but to learn different things and that __for example we have time for exchanges. Finally, to have a__ a different pedagogy and to study in a way that is perhaps a little more interesting” (E11)”.*

#### Being more productive

Finally, participants mentioned aspects of productivity in one’s work. Medical students expected to improve their performance. Indeed, with humor and authenticity, some of them underlined their need to get better in order to be more productive. The challenge of medica studies and the competition was evoked, and some reported doubts to be able to do it. Meditation training could then represent a mean to enhance personal productivity.

*“and actually what I was really interested in was the productivity (laughs) (…) __ I don’t know. I thought you’re able to multitask and be a better doctor(E5)”.*

### Caring for my health

If the motivation turned towards others, echoing their function as future doctors, was often the first one mentioned by students, the motivation turned towards their own health was also rapidly put forward. Respondents produced a higher quantity of verbal material relating to this theme. It includes three sub themes: stress reduction and well-being improvement, taking time for oneself, and improving self-compassion.

#### Stress reduction, emotion regulation and well-being improvement

Participants reported being motivated by stress reduction. They spoke about their anxiety and reported sometimes it had a strong impact on their daily life. Fatigue, lack of sleep or concentration issues were mentioned as elements related to stress, they wished to mitigate with mindfulness practice.

*“I told myself that I was also doing it for myself. Because I realized not long ago that when it’s time to go to sleep, well, for me it’s more of an ordeal than anything else. And so I don’t sleep much and so I want to live it as a good moment and sleep well like everybody else. Yeah, to spend some good moments alone with myself(E26)”.*

Participants also mentioned the difficulty managing emotions, as an element often invading and impacting the quality of life but also the care relationship.

*I wanted to be able to better manage my emotions, to better take stock of them in order to be able to finally (speeding up of the voice) better take care of the patients(E3)”.*

At this stage of the program, they evoked the reciprocal link between their health and their role as caregiver. Participants spontaneously linked their responsibility to take care of themselves with their duty to take care of their patient.

*“If I can understand myself better, I can understand my patients better (E27)”.*

#### Take time for oneself

Another element of motivation seems to be simply the need to take some time without having a specific goal. Thus, enjoying time during the week to do something for themselves seemed to be an important motivator. Again, they recall the intense academic routine and a need to slow down. Participants expressed their wish to cultivate another relation with time, learn to anticipate less, and live more intensely in the present moment.

*“to take the time to do things more slowly, to settle down, not to chain actions, thinking about what we were going to do afterwards etc. That’s what came to me first (E1)”.*

#### Improving self-compassion

Taking care of oneself was evoked by some participants as an act of love, tenderness, and friendship. The evocation of this motivation emerged as a “surprising” need, giving way to emotions underlined in the non-verbal language “*voice trembling(E28)”.* In response to the demanding nature of medical education, participants spoke of the need to learn to be more tolerant toward themselves. They thus reported a need to cultivate self-compassion. Some of the students had already been exposed to mindfulness practice and knew they would find in meditation.

“*… a moment where I find I can allow myself to feel love for myself, it feels good(E28)”.*

### A quest for meaning

The content of the last theme underlies both previously presented themes, namely the care of self and others. This theme describes the need to find a greater engagement to continue learning, living, and caring along the medical studies and career. To train in mindfulness would support this quest, provide meaning to care and to life in general.

#### Cultivate mindfulness

In fact, many students had already heard of meditation, sometimes even had been meditating through yoga or other mind-body practices such as apps. They expressed a wish to deepen and pursue their experience. The desire to continue and integrate this practice into daily life as is proposed in MBSR practice was important.

*“Because I knew that I had done meditation before and it felt good and I wanted to include it in my life in general and I couldn’t do it on my own so__ having some help is good(E9)”.*

#### Meaning of care

Through this quest for meaning, the quest for the meaning of the nursing profession is put forward. Indeed, as if the motivation to be there was to find an answer to the fundamental question of why I become a doctor, and how I wish to heal?

I also wonder if the fact that I take care of others is not purely selfish, to avoid thinking about myself. And so I want to help others because I want to help them and not only to escape from my head to head with myself(E27).

I’ve always had a goal, always succeeded, to do this, that, that, so I move forward with a goal every time I’m given one and in fact I’m coming to the end of my studies... And so I think it’s important to sit down and say to myself what I want(E22).

#### Meaning of life

For others, the questions raised were even more profound and dealt with the meaning of life itself. Participants mentioned a need to enrich their spiritual life. It seemed that in mindfulness practice they wished to develop a form of secular spirituality. They made connections between concrete aspects of training and some religious activities: using silence, fostering community, and developing goodness and non-judgment, and underlined their desire to cultivate it outside religion.

*“This question of doing actions that are right; so a need to have this guidance to really help me(E26)”.*

*“I heard the word conviction that spoke to me, it’s more, I know it a little bit from experience that it feels good and there was a kind of certainty actually that surprised me(E26)”.*

## Discussions

In a qualitative analysis of verbal material collected in the first session of MBSR in 29 students, we found three broad categories of motivations. The first was a motivation to improve one’s own medical education and the physician’s role. It included motivations such as the need to improve interpersonal skills or other skills oriented toward a more integrative medicine or being attracted to an experiential learning framework. The second describes the need to take care of one’s own health, through reducing stress and enhancing self-compassion. The third refers to a drive of searching for meanings, of care and life. We will first discuss the coherence between the motivations of the students, their needs and the evidence available, then the new elements of understanding.

### Concordance with literature

The first striking aspect of the results is the fit between the perceived motivations of students and the objectives of the teaching as described in the scientific literature. This is particularly true with expectancies regarding mental health. In fact, empirical studies have demonstrated that in medical students, MBSR was beneficial to reduce anxiety, perceived stress and overall psychological distress [14, 32–34]. De Vibe et al. looked directly at self-reported subjective well-being and found a significant increase following MBSR with a long-term effect at six year [35,36]. Looking at self-compassion, Erogul et al. measured self-compassion using the Self-Compassion Scale, which has been associated with positive psychological health and found significant increases post-intervention and at six-month follow-up. In fact, previous research has suggested that when physicians feel well, they are best able to meaningfully connect with and care for patients (Thomas LR, 2018). Training in mindfulness leads to increases in decentering, emotion regulation, self-compassion, enhanced working memory which then lead to positive health outcomes [37]. Participants of the present sample appeared to be aware of this process and wished to take care of themselves to better take care of others. Other elements of our results underline their need to improve their skills in relation to the care relationship, which may refer to communication or empathy skills. In addition to their benefits for well-being, studies have suggested that MBIs could indeed facilitate professional skills such as resilience and the ability to set priorities and boundaries [33,38]. Although evidence for effects on medical student interpersonal skills are less clear, some results have suggested that mindfulness could favor empathy skills [39,40]. In their qualitative study, Solhaug et al. found that students participating in MBSR described patient encounters with more compassion [41]. In a two-group pilot-test, researchers have found encouraging results suggesting MBSR would yield a better understanding of one’s own emotion, and empathy [42]. However such research is still scarce and interpersonal competencies are mostly explored with self-report, which bear high risks of bias in this field. These results highlight the needs of students to acquire additional interpersonal skills that MBSR may not fully address. However, they shared the need to learn as a group. In fact, the group-based setting of an MBI, which promoted shared experience and peer support, also appeared beneficial in qualitative study for well-being [43,44]. Specifically, general practitioners noticed that sharing experiences with peers helped them to deal with stressful events by providing reassurance that they were not alone in their feelings [43].

The motivation to engage in a mindfulness course to cultivate a sense of care also seemed congruent with the development of curricula that offer a humanistic and holistic approach to medicine as is the case in Yale School of Public Health [45]. These curricula incorporate mindfulness as a central component. In fact, students’ explicit need is to acquire advanced skills to be able to adopt more humanistic models such as the mindful practitioner [46–48]. These curricula have consistently attempted to meet contemporary patients’ needs [49] and appear to be consistent with the needs of students. These are being slowly integrated into medical schools around the world but are not yet part of the official medical training in France.

Another interesting result in line with literature on mindfulness purpose concerned the motivation to cultivate one’s meaning of life. These results refer to the wisdom of mindfulness practice [19,50]. Consistently, in its broadest sense, mindfulness refers to a spiritual path for cultivating well-being and alleviating suffering [50]. Students seemed to be keenly aware of this aspect as it was a strong motivation to participate. Some people in the group had already meditated and could be the ones who shared this motivation. However, in the context of medicine, this element may be seen as new to medical university leaders. The term spirituality is not used much in medicine and medical curricula, except for specific domains like clinical ethics or palliative care [51]. It may also appear scary in some cultures [51]. According to the holistic health definition given by Svalastog et al., *“health is a relative state in which one is able to function well physically, mentally, socially, and spiritually to express the full range of one’s unique potentialities in the environment in which one lives”*, spiritual care can be considered an essential component of medical care [52]. The spiritual dimension can support uncertainty related to care, or to the human condition in the face of illness or finitude [53]. In our perspective, the link between MBIs and spirituality in the context of medical education and how it contributes to care should be reconsidered and evaluated.

### New elements of understanding

Participants from the present study mentioned the use of MBSR to enhance their performance *(training to gain productivity*). This search for increased productivity is actually at odds with the original spirit of mindfulness, more centered on the spiritual path for cultivating well-being and alleviating suffering [50]. This highlights the risk to instrumentalize mindfulness that may threaten the process itself. Research from highly competitive environments such as the corporate world [54] or in athletes [55], have found that motivations other than productivity help participants gain in mindfulness skills, be more engaged, and become productive and effective. Positive benefits of mindfulness over time will happen and very probably depend on motivations towards the process and well-being-related outcomes. For instance, recent neuroscience results have found arguments in favor of enhanced learning skills induced by repeated meditation with identified anatomic neuroplasticity and gray matter concentrations in brain regions involved in learning [56,57], and suggests the influence of mindfulness on learning skills. Medical students have a performance goal through successful completion of exams. Recent non-randomized controlled trials have looked at this performance criterion in medical students who have participated in MBIs [58]. They showed that beneficial effects on scholarly success were transient and only detectable at completion of the intervention. [SS1] Disentangling personal motivations, from managing stress to being more acute cognitively should be researched in more systematic ways.

Time was another important topic brought by participants. While some studies of medical students indicate that lack of time is seen as a barrier to participation in meditation programs [44,59,60] our work showed it is a motivating factor. Previous experiences have found that students may feel guilty about taking time for themselves and spending less time on their work. In contrast, participants expressed that taking time for their health was directly related to their role as a physician. The motivation of medical students to care for themselves in order to care for others may be related to an awareness of the “interconnectedness” between people [61]. Interconnectedness is rooted in Buddhist meditation practice and describes the interdependent nature of all phenomena in the world, which implies that the appearance of all things is conditioned by the appearance of others [19]. The motivation to take care of oneself could be considered as a new need inherent to care. It would be interesting to clarify this aspect and better understand its consequences in care.

### Limitations

We should recognize the limitations of this work. First, the time allowed to each participant was limited and the format did not allow for each student to develop their thoughts with details. Second, some participants may have been reluctant to share personal feelings or motives as they were meeting for the first time. It is thus possible that transcripts contain the views of those who were sufficiently asserted to speak up. Other data collection methods should probably be used in the future (e.g. individual interviews or open surveys). Some students, even if they had never participated in an MBSR program, had already meditated. This may have influenced our results related to congruence, although to a small extent as these were a small proportion.

## Conclusion

In a qualitative analysis of students’ motivations to participate in MBSR class we found a high consistency with the existing evidence on the effect of mindfulness concerning self-care, the development of humanistic medical skills, and the meaning of care. Some findings point to the risk of instrumentalizing mindfulness to use it as a tool to enhance one’s efficiency. One of the main motivations of medical students was to take care of themselves, in order to be able to take care of others, showing a paradigm shift for the new generation of physicians. Studying students’ motivations is essential to adjusting curricula.

## Electronic supplementary material

Below is the link to the electronic supplementary material.

**Additional file 1**: Appendix 1 and 2

## Data Availability

The datasets used and/or analysed during the current study available from the corresponding author on reasonable request.

## References

[1] Dyrbye LN, Thomas MR, Massie FS (2008). Burnout and suicidal ideation among U.S. medical students. Ann Intern Med.

[2] Rotenstein LS, Ramos MA, Torre M (2016). Prevalence of Depression, depressive symptoms, and suicidal ideation among medical students: a systematic review and Meta-analysis. JAMA.

[3] Worsley JD, Pennington A, Corcoran R (2022). Supporting mental health and wellbeing of university and college students: a systematic review of review-level evidence of interventions. Carrà G. ed PLOS ONE.

[4] Fond G, Bourbon A, Lançon C (2018). Psychiatric and psychological follow-up of undergraduate and postgraduate medical students: prevalence and associated factors. Results from the national BOURBON study. Psychiatry Res.

[5] Kansoun Z, Boyer L, Hodgkinson M, Villes V, Lançon C, Fond G (2019). Burnout in french physicians: a systematic review and meta-analysis. J Affect Disord.

[6] Rolland F, Hadouiri N, Haas-Jordache A (2022). Mental health and working conditions among french medical students: a nationwide study. J Affect Disord.

[7] Hofmann SG, Sawyer AT, Witt AA, Oh D (2010). The effect of mindfulness-based therapy on anxiety and depression: a meta-analytic review. J Consult Clin Psychol.

[8] Zoogman S, Goldberg SB, Hoyt WT, Miller L (2015). Mindfulness interventions with youth: a Meta-analysis. Mindfulness.

[9] Dobkin PL, Hutchinson TA (2013). Teaching mindfulness in medical school: where are we now and where are we going?. Med Educ.

[10] Dyrbye LN, Sciolla AF, Dekhtyar M (2019). Medical School strategies to address Student Well-Being: A National Survey. Acad Med.

[11] Thomas LR, Ripp JA, West CP. Charter on Physician Well-being. JAMA. 2018;29. 10.1001/jama.2018.1331. 10.1001/jama.2018.1331 29596592

[12] Wasson LT, Cusmano A, Meli L (2016). Association between Learning Environment Interventions and Medical Student Well-being: a systematic review. JAMA.

[13] Polle E, Gair J. Mindfulness-based stress reduction for medical students: a narrative review. Can Med Educ J. 2021;31. 10.36834/cmej.68406. 10.36834/cmej.68406 PMC8105581 33995723

[14] McConville J, McAleer R, Hahne A (2017). Mindfulness training for Health Profession Students—The Effect of Mindfulness Training on Psychological Well-Being, Learning and clinical performance of Health Professional students: a systematic review of Randomized and non-randomized controlled trials. EXPLORE.

[15] West CP, Dyrbye LN, Erwin PJ, Shanafelt TD (2016). Interventions to prevent and reduce physician burnout: a systematic review and meta-analysis. The Lancet.

[16] Dyrbye LN, Thomas MR, Shanafelt TD (2006). Systematic review of Depression, anxiety, and other indicators of psychological distress among U.S. and Canadian Medical Students. Acad Med.

[17] Kunzler AM, Helmreich I, Chmitorz A, et al. Psychological interventions to foster resilience in healthcare professionals. Cochrane Developmental, Psychosocial and learning problems Group. ed Cochrane Database Syst Rev. 2020;2020(7). 10.1002/14651858.CD012527.pub2. 10.1002/14651858.CD012527.pub2 PMC8121081 32627860

[18] Carmody J, Baer RA, Lykins LB, Olendzki E (2009). An empirical study of the mechanisms of mindfulness in a mindfulness-based stress reduction program. J Clin Psychol.

[19] Husgafvel V, THE (2018). Contemp Buddhism.

[20] Williams JMG, Kabat-Zinn J (2011). Mindfulness: diverse perspectives on its meaning, origins, and multiple applications at the intersection of science and dharma. Contemp Buddhism.

[21] Kabat-Zinn J. Full catastrophe living (revised Edition): using the Wisdom of your body and mind to face stress, Pain, and illness. Bantam Books; 2013.

[22] Shapiro SL, Carlson LE, Astin JA, Freedman B (2006). Mechanisms of mindfulness. J Clin Psychol.

[23] Martin AJ (2004). School motivation of boys and girls: differences of degree, differences of kind, or both?. Aust J Psychol.

[24] Kabat-Zinn J, Hanh TN. Full catastrophe living: using the wisdom of your body and mind to face stress, pain, and illness. Delta; 2009.

[25] Ludwig DS, Kabat-Zinn J (2008). Mindfulness in medicine. JAMA.

[26] Kabat-Zinn J (2003). Mindfulness-based interventions in context: past, present, and future. Clin Psychol Sci Pract.

[27] Kabat-Zinn J, Meleo-Meyer F, Koerbel L, Santorelli S, MBSR Curriculum Guide. 2017. https://lotheijke.com/wp-content/uploads/2020/11/8-week-mbsr-authorized-curriculum-guide-2017.pdf

[28] Braun V, Clarke V (2006). Using thematic analysis in psychology. Qual Res Psychol.

[29] Paillé P, Mucchielli A. *L’analyse Qualitative En Sciences Humaines et Sociales* 4e édition.; 2016.

[30] Paillé P. Les conditions de l’analyse qualitative: Réflexions autour de l’utilisation des logiciels. *SociologieS*. 2011;6. 10.4000/sociologies.3557.

[31] Tong A, Sainsbury P, Craig J (2007). Consolidated criteria for reporting qualitative research (COREQ): a 32-item checklist for interviews and focus groups. Int J Qual Health Care.

[32] Irving JA, Dobkin PL, Park J (2009). Cultivating mindfulness in health care professionals: a review of empirical studies of mindfulness-based stress reduction (MBSR). Complement Ther Clin Pract.

[33] Scheepers RA, Emke H, Epstein RM, Lombarts KMJMH (2020). The impact of mindfulness-based interventions on doctors’ well‐being and performance: a systematic review. Med Educ.

[34] van Dijk I, Lucassen PLBJ, Akkermans RP, van Engelen BGM, van Weel C, Speckens AEM (2017). Effects of Mindfulness-Based stress reduction on the Mental Health of Clinical Clerkship students: a cluster-randomized controlled trial. Acad Med.

[35] de Vibe M, Solhaug I, Tyssen R (2013). Mindfulness training for stress management: a randomised controlled study of medical and psychology students. BMC Med Educ.

[36] de Vibe M, Solhaug I, Rosenvinge JH, Tyssen R, Hanley A, Garland E. Six-year positive effects of a mindfulness-based intervention on mindfulness, coping and well-being in medical and psychology students; Results from a randomized controlled trial. Moitra E, ed. *PLOS ONE*. 2018;13(4):e0196053. doi:10.1371/journal.pone.0196053 10.1371/journal.pone.0196053 PMC5916495 29689081

[37] Guendelman S, Medeiros S, Rampes H. Mindfulness and emotion regulation: insights from Neurobiological, Psychological, and Clinical Studies. Front Psychol. 2017;8. 10.3389/fpsyg.2017.00220. 10.3389/fpsyg.2017.00220 PMC5337506 28321194

[38] Costa-Drolon E, Verneuil L, Manolios E, Revah-Levy A, Sibeoni J (2021). Medical students’ perspectives on Empathy: a systematic review and metasynthesis. Acad Med.

[39] Lamothe M, McDuff P, Pastore YD, Duval M, Sultan S (2018). Developing professional caregivers’ empathy and emotional competencies through mindfulness-based stress reduction (MBSR): results of two proof-of-concept studies. BMJ Open.

[40] Shapiro SL, Schwartz GE, Bonner G (1998). Effects of mindfulness-based stress reduction on medical and premedical students. J Behav Med.

[41] Solhaug I, Eriksen TE, de Vibe M (2016). Medical and psychology student’s experiences in learning mindfulness: benefits, Paradoxes, and Pitfalls. Mindfulness.

[42] Lamothe M, Rondeau É, Malboeuf-Hurtubise C, Duval M, Sultan S (2016). Outcomes of MBSR or MBSR-based interventions in health care providers: a systematic review with a focus on empathy and emotional competencies. Complement Ther Med.

[43] Beckman HB, Wendland M, Mooney C (2012). The impact of a program in Mindful Communication on Primary Care Physicians. Acad Med.

[44] Verweij H, van Ravesteijn H, van Hooff MLM, Lagro-Janssen ALM, Speckens AEM (2018). Does Mindfulness Training enhance the Professional Development of residents? A qualitative study. Acad Med.

[45] Nawaz H, Via CM, Ali A, Rosenberger LD, Project ASPIRE (2015). Incorporating Integrative Medicine into Residency Training. Am J Prev Med.

[46] Epstein RM, Mindful Practice (1999). JAMA.

[47] Hassed C. The Art of Introducing Mindfulness into Medical and Allied Health Curricula. 2021:1909–1919.

[48] Hutchinson TA. Relationship in clinical practice. In: *whole person care*. Springer Int Publishing. 2017;37–44. 10.1007/978-3-319-59005-9_5.

[49] Gannotta R, Malik S, Chan AY, Urgun K, Hsu F, Vadera S. Integrative Medicine as a vital component of Patient Care. Cureus. 2018;4. 10.7759/cureus.3098. 10.7759/cureus.3098 PMC6173273 30338174

[50] Bodhi B (2011). What does mindfulness really mean? A canonical perspective. Contemp Buddhism.

[51] Wenham J, Best M, Kissane DW (2021). Systematic review of medical education on spirituality. Intern Med J.

[52] Svalastog AL, Donev D, Jahren Kristoffersen N, Gajović S (2017). Concepts and definitions of health and health-related values in the knowledge landscapes of the digital society. Croat Med J.

[53] Koper I, Pasman HRW, Schweitzer BPM, Kuin A, Onwuteaka-Philipsen BD (2019). Spiritual care at the end of life in the primary care setting: experiences from spiritual caregivers - a mixed methods study. BMC Palliat Care.

[54] Hyland PK, Lee RA, Mills MJ (2015). Mindfulness at work: a New Approach to improving individual and organizational performance. Ind Organ Psychol.

[55] Nien JT, Wu CH, Yang KT (2020). Mindfulness training enhances endurance performance and executive functions in athletes: an event-related potential study. Neural Plast.

[56] Boccia M, Piccardi L, Guariglia P (2015). The meditative mind: a Comprehensive Meta-Analysis of MRI Studies. BioMed Res Int.

[57] Pernet CR, Belov N, Delorme A, Zammit A (2021). Mindfulness related changes in grey matter: a systematic review and meta-analysis. Brain Imaging Behav.

[58] Lampe LC, Müller-Hilke B (2021). Mindfulness-based intervention helps preclinical medical students to contain stress, maintain mindfulness and improve academic success. BMC Med Educ.

[59] Aherne D, Farrant K, Hickey L, Hickey E, McGrath L, McGrath D (2016). Mindfulness based stress reduction for medical students: optimising student satisfaction and engagement. BMC Med Educ.

[60] Razzaque R, Wood L (2016). Exploration of the effectiveness and acceptability of a Professional Mindfulness Retreat for Psychiatrists. Mindfulness.

[61] Kalra S, Priya G, Grewal E (2018). Lessons for the Health-care Practitioner from Buddhism. Indian J Endocrinol Metab.

