# Supplementary material for: Understanding students’ motivations for participating in a mindfulness course: a qualitative analysis of medical students’ views
Source: BMC Complement Med Ther. 2023 May 20;23:163. doi: 10.1186/s12906-023-03949-2 (PMC10199565; doi:10.1186/s12906-023-03949-2)
Supplement: Supplementary file 1 — Additional file 1: Appendix 1 and 2 [file 12906_2023_3949_MOESM1_ESM.docx]

**Appendix.1: team reflexivity**

The first author (CB) is a doctor and a mindfulness teacher. In this work, she embodies the dual posture of teacher-researcher, a choice that may seem like a limitation, but which is meaningful here. Indeed, qualitative researchers require the ability to go back and forth between reality and thoughts and suspend critical judgment. This process is very close to what happens during mindfulness training [(Bitbol, 2019)](https://www.zotero.org/google-docs/?ohKJ3d). In fact, we considered in the study that training in mindfulness helps the first author to reinforce their qualitative researcher’s skills: tolerate the uncertainty, know how to read and verbalize her own affects, accept complexity and subjectivity, and work with a team [(Devereux, 2012)](https://www.zotero.org/google-docs/?EiJPsf). Mostly, for three years, CB developed expertise in teaching MBSR to medical students. Her background as a doctor as well might help students to settle with authenticity in the D&I process. Mostly, these multiple ways involvement helped her to observe and detach from its beliefs. On the other hand, the team members SS and FZ have been involved in much research on cognitive behavior and psychosocial dimensions in healthcare including emotional competencies. Although, to ensure validity to data analyses, researchers worked as a team with other psychologists (HG and LR) and researchers (MAP). HG and CB conducted separate analyses of the verbatim transcript and compared them afterwards. Two other researchers triangulated the analysis LR and MAP. LR has been a postdoc working in psychology and has been supervising many qualitative studies using inductive thematic analysis. She brings a structured lay vision to interpretations. MAP is an adolescent and young people psychiatrist and expert in qualitative research with a large involvement in medical education. MAP followed the study and gave her feedback according to data analyses. Other of our authors have been major figures in the development in the field of psychiatry and mental health. The final organization emerged from the collaborative work of authors. Overall, our team was guided by an evidence-based approach. As a consequence, the literature and the components of existing data guided our preconceptions that participants would mainly attend to the class to reduce stress and anxiety [(Amri & Haramati, 2010; Dobkin & Hutchinson, 2013; Ludwig & Kabat-Zinn, 2008; van Dijk et al., 2017)](https://www.zotero.org/google-docs/?zH8mlU).

**Appendix.2 : Theme, sub-themes, code and verbatim illustrations of perceived motivation by medical students that participate in MBSR.**

| Theme/ Sub-themes | code | Verbatim illustration |
| --- | --- | --- |
| **Medical education and the physician’ s role** | **Listening to others**  **holistic approach**  **Rupture**  **Alternative pedagogy**  **Learning**  **Group practice**  **Performance**  **Improvement**  **Productivity** |  |
| Improving interpersonal skills |  | *“I thought I liked being in a relationship, a real relationship with the patients and the people we were talking to(E7)”.*  *“I hope to be able to*  *find the right words a little more and to have a sympathetic ear(E24)”.*  *"Why I'm really really here, I thought it might teach me to understand my emotions(E4)”.* |
| Learning complementary skills |  | *“I like to test everything I offer my patients so I find it interesting to know what we're talking about, so that was the primary reason(E21)“.* |
| Attracted by another approach |  | *“So there you go, as I was saying to M..., I was excited to do this (laughs)(E1)”.*  *“I wanted to __ not just to learn something but that __ or to learn things but to learn different things and that __for example we have time for exchanges. Finally, to have a__ a different pedagogy and to study in a way that is perhaps a little more interesting (E11)”.* |
| Being more productive |  | *“and actually what I was really interested in was the productivity (laughs) (...) __ I don't know. I thought you're able to multitask and be a better doctor (E5)”.* |
| **Caring for my health** | **Internal fog**  **Control**  **Pressure**  **Emotion**  **Anxiety**  **Perspective taking**  **Resources**  **Relaxation**  **take the time**  **Love of oneself**  **Opening up**  **Awareness** |  |
| Stress reduction, emotion regulation and well-being improvement |  | *(speeding up of the voice) better take care of the patients (E3)*  *“ If I can understand myself better, I can understand my patients better(E27)”.* |
| Take time for oneself |  | *“to take the time to do things more slowly, to settle down, not to chain actions, thinking about what we were going to do afterwards etc. That's what came to me first(E1) ».* |
| Improving self-compassion |  | “*… a moment where I find I can allow myself to*  *feel love for myself, it feels good(E28)”.* |
| **A quest for meaning** | **Adventure**  **process**  **Values**  **Commitment**  **Lack of certainty**  **Purpose**  **Existence**  **worldwide**  **path**  **hope**  **trust** |  |
| Cultivate mindfulness |  | *“Because I knew that I had done meditation before and it felt good and I wanted to include it in my life in general and I couldn't do it on my own so__ having some help is good(E9)”.* |
| Meaning of care |  | *“I also wonder if the fact that I take care of others is not purely selfish, to avoid thinking about myself. And so I want to help others because I want to help them and not only to escape from my head to head with myself(E27).”*  *“I've always had a goal, always succeeded, to do this, that, that, so I move forward with a goal every time I'm given one and in fact I'm coming to the end of my studies... And so I think it's important to sit down and say to myself what I want(E22).”* |
| Meaning of life |  | *“This question of doing actions that are right; so a need to have this guidance to really help me(E26)”.*  *“I heard the word conviction that spoke to me, it's more, I know it a little bit from experience that it feels good and there was a kind of certainty actually that surprised me(E26)”.* |
